# Derivation and Validation of an Optimal Neutrophil Gelatinase-Associated Lipocalin Cutoff to Predict Stage 2/3 Acute Kidney Injury (AKI) in Critically Ill Children

**DOI:** 10.1016/j.ekir.2024.05.010

**Published:** 2024-05-15

**Authors:** Stuart L. Goldstein, Ayse Akcan-Arikan, Natasha Afonso, David J. Askenazi, Abby M. Basalely, Rajit K. Basu, Hostensia Beng, Julie C. Fitzgerald, Katja Gist, Sarah Kizilbash, David Kwiatkowski, Christopher W. Mastropietro, Shina Menon, Megan SooHoo, Avram Z. Traum, Christopher A. Bird

**Affiliations:** 1 Cincinnati Children’s Hospital Medical Center, University of Cincinnati College of Medicine, Cincinnati, Ohio, USA; 2 University of Cincinnati College of Medicine, Cincinnati, Ohio, USA; 3 Baylor College of Medicine/Texas Children’s Hospital, Houston, Texas, USA; 4 Children’s of Alabama, University of Alabama at Birmingham, Birmingham, Alabama, USA; 5 Cohen Children’s Medical Center at Northwell Health, New York, USA; 6 Lurie Children’s Hospital, Northwestern University Feinberg School of Medicine, Illinois, Chicago, USA; 7 East Carolina University Brody School of Medicine, Greenville, North Carolina, USA; 8 Children’s Hospital of Philadelphia, Philadelphia, Pennsylvania, USA; 9 University of Minnesota Children’s Hospital Minneapolis, Minnesota, USA; 10 Lucille Packard Children’s Hospital Stanford University, Palo Alto, California, USA; 11 Riley Hospital for Children at Indiana University Health University of Indiana School of Medicine, Indianapolis, Indiana, USA; 12 Seattle Children’s Hospital, Seattle, Washington, USA; 13 Children’s Hospital of Colorado, Aurora, Colorado, USA; 14 Boston Children’s Hospital, Boston, Massachusetts, USA; 15 BioPorto Diagnostics, Copenhagen, Denmark, Europe

**Keywords:** acute kidney injury, children, neutrophil gelatinase-associated lipocalin, NGAL

## Abstract

**Introduction:**

Acute kidney injury (AKI) defined by changes in serum creatinine (SCr), or oliguria is associated with increased morbidity and mortality in children who are critically ill. We derived and validated a clinical cutoff value for urine neutrophil gelatinase-associated lipocalin (NGAL), in a prospective multicenter study of children who were critically ill. We report the clinical performance of urine NGAL (uNGAL) to aid in pediatric AKI risk assessment.

**Methods:**

Eligible subjects were aged ≥ 90 days to < 22 years, admitted to an intensive care unit (ICU), and had 1 or more of the following: mechanical ventilation, vasoactive medication administration, solid organ or bone marrow transplantation, or hypotension within 24-hours of admission. uNGAL was assessed within 24-hours of admission. The primary outcome was SCr-based stage 2/3 AKI presence at 48- to 72-hours.

**Results:**

Twenty-five (12.3%) derivation study patients had stage 2/3 AKI at 48- to 72-hours. uNGAL concentration of 125 ng/ml was the optimal cutoff. Forty-seven (9.1%) validation study patients had stage 2/3 AKI at 48- to 72-hours. The area under the curve of a receiver operator characteristics curve (AUC-ROC) for uNGAL performance was 0.83 (95% confidence interval [CI]: 0.77–0.90). Performance characteristics were sensitivity 72.3% (95% CI: 57.4%–84.4%), specificity 86.3% (95% CI: 82.8%–89.3%), positive predictive value 34.7% (95% CI: 28.5%–41.5%), and negative predictive value 96.9% (95% CI: 95.1%–98.0%).

**Conclusion:**

These prospective, pediatric, multicenter studies demonstrate that uNGAL in the first 24-hours performs very well to predict Kidney Disease Improving Global Outcomes (KDIGO) stage 2/3 AKI at 48- to 72-hours into an ICU course. We suggest that a uNGAL cut point of 125 ng/ml can aid in the risk assessment for stage 2/3 AKI persistence or development.

## Graphical abstract

**Figure:**
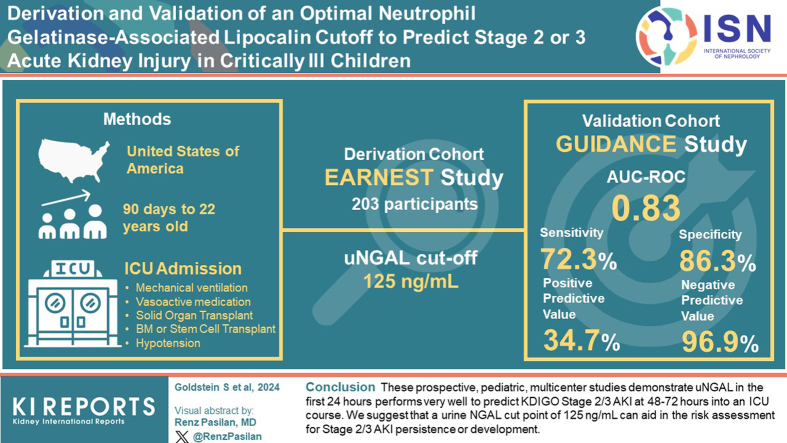

See Commentary on Page 2329

AKI comprises a heterogeneous set of underlying conditions defined by an abrupt loss of kidney function, currently diagnosed by increase in SCr or a specific degree and duration of oliguria. Since publication of the unified, standardized, multidimensional KDIGO AKI diagnostic staging criteria,^1^ multicenter studies demonstrate especially strong associations with KDIGO stage 2 or 3 AKI (stage 2/3 AKI) and poor patient outcomes in infants, children, and adults who are critically ill.^2^, ^3^, ^4^, ^5^, ^6^ Although these epidemiological insights have been fundamental to increase awareness of the importance of AKI and its short- and long-term consequences, the functional SCr and oliguria criteria are late and undifferentiating markers of kidney injury.^7^

As a result, extensive basic, translational, and clinical research efforts have focused on discovery and validation of urinary proteins to identify subclinical kidney injury before SCr changes or oliguria and to differentiate true tubular stress and damage from purely functional changes.^7^^,^^8^ NGAL, a 25k Da protein and one of the most highly upregulated gene products induced by tubular injury, has been the subject of > 600 publications in the human AKI literature and at least 5 systematic reviews in the last 5 years.^9^, ^10^, ^11^, ^12^, ^13^ NGAL demonstrates consistent good-to-excellent performance to predict creatinine/urine output-based AKI earlier and identify subclinical AKI in neonates, infants, children, and young adults in various clinical settings including cardiac surgery, abdominal surgery, and in critical illness.^14^, ^15^, ^16^, ^17^ Despite the abundance of research describing uNGAL performance in the pediatric arena summarized in a recent meta-analysis of 38 studies comprising 4658 patients,^13^ widespread clinical practice adoption of NGAL has yet to occur. One potential reason for the lack of clinical adoption is the absence of a community-wide accepted reference standard uNGAL reference cutoff concentration to codify performance characteristics to achieve clearance by the US Food and Drug Administration for marketing this diagnostic test.

To address this gap, we undertook 2 prospective, multicenter studies to assess the performance of uNGAL to predict KDIGO stage 2/3 AKI in children who were critically ill admitted to a pediatric intensive care unit or pediatric cardiac intensive care unit. We hypothesized that we could derive (first study) and then validate (second study) the same uNGAL concentration cutoff to demonstrate excellent predictive performance with focus on optimizing sensitivity. Data for the validation study comprised the clinical study report for a Food and Drug Administration marketing application.

## Methods

### Study Design and Participants

EARNEST and GUIDANCE were multicenter prospective studies designed to determine the cutoff and validation of the urinary ProNephro AKI (NGAL) assay (BioPorto Diagnostics, Needham, MA). The first (cutoff derivation) study, the Establishment of the BioPorto Diagnostics NGAL Test Clinical Cut-off Value for Risk Assessment of Moderate to Severe AKI in a Pediatric Population (EARNEST) enrolled 257 subjects from 6 US institutions (Cincinnati Children’s Hospital Medical Center, Seattle Children’s Hospital, Children’s Hospital Colorado, Texas Children’s Hospital, Lucille Packard Children’s Hospital at Stanford, and Children’s Healthcare of Atlanta at Eggleston) from June 2020 through June 2021. The second (cutoff validation) study, NGAL usage in determining AKI risk in critically ill children (GUIDANCE), enrolled 660 subjects at the same institutions in EARNEST (with the exception of Children’s Healthcare of Atlanta) and additional sites of Riley Hospital for Children at Indiana University Health, Children’s of Alabama, Brody School of Medicine at East Carolina University, University of Minnesota Children’s Hospital, Virginia Commonwealth University, Arkansas Children’s Hospital, Cohen Children’s Medical Center, Lurie Children’s Hospital, Boston Children’s Hospital, and Children’s Hospital of Philadelphia from December 2020 through August 2022. Each institution received approval from local institution review board for each study separately before subject enrollment. Written informed consent from parent/caregiver was required for sample collection and use of data for analysis. Some site institution review boards allowed for delayed consent to allow for urine sample collection, given the acute nature of AKI and compressed time window, whereas other institution review boards did not. Samples and data were discarded in instances in which delayed consent was not obtained.

### Eligibility Criteria

Eligible subjects were between ≥ 90 days and < 22 years of age and admitted to a pediatric intensive care unit or pediatric cardiac intensive care unit. Subjects had to meet 1 of the following leading to admission or occurring within the first 24-hours of ICU stay as eligibility criteria: mechanical ventilation, vasoactive medication administration, history of solid organ transplantation (kidney transplantation included only if > 3 months before), history of bone marrow/stem cell transplantation, or hypotension, defined as having received ≥ 40 ml/kg of resuscitative fluid in pre-ICU within 6 hours before or in first 12 hours of ICU admission. The main exclusion criteria included receipt of renal replacement therapy in the first 24-hours of ICU admission, documented urinary tract infection, known chronic kidney disease stage 4 or 5, or known congenital anomaly of the kidney or urinary tract. A complete list of exclusion criteria is listed in Supplementary Table S1. Subjects whose conditions were not evaluated included screen-failed subjects not meeting inclusion/exclusion criteria, subjects who were withdrawn, or subjects excluded from analysis per protocol requirements including missing urine or SCr values, as well as improper handling, storage, processing, or insufficient volume of samples.

### Procedures

#### Sample and data collection

Urine samples were obtained from enrolled subjects using an indwelling bladder catheter, urine collection device, or clean catch per standard of care once from 0- to 24-hours after ICU admission. Urine samples were kept at room temperature for up to 4-hours, or up to 48-hours at 2 °C to 8 °C, before being processed and frozen. Samples were transferred to a clean tube and centrifuged at 1500 g for 15 minutes. Immediately after centrifugation, samples were aliquoted into 1 ml aliquots, and frozen at −70 °C or colder. Samples were stored at −70 °C or colder until they were batched shipped on dry ice to Cincinnati Children’s Hospital Medical Center, Division of Nephrology and Hypertension, where all samples were then stored and catalogued in a biorepository.

Blood samples for SCr measurement were obtained as part of standard of care. Although the clinical critical care teams were aware of the occurrence of each study, sample collection for SCr was not mandated for clinical purposes-research samples were obtained when clinical samples were not ordered. Clinicians were blinded to the uNGAL results.

All relevant clinical data including patient demographics, prior health history, and reason for ICU admission were collected from the hospital records and stored using electronic case-report forms in an anonymized password-protected database (iMednet EDC platform, Mednet, MN).

### NGAL Measurements

Urine samples were shipped from Cincinnati Children’s Hospital Medical Center in batches and analyzed retrospectively at an independent testing site (Yale University, Yale New Haven Hospital). Concentrations of NGAL were determined with the ProNephro AKI (NGAL) test (BioPorto Diagnostics Inc, Needham, Manchester), the device under investigation, on a Roche cobas c 501 analyzer (Roche Diagnostics, Indianapolis, IN). Within run and between run coefficients of variation were tested before analyzing the samples; within run coefficients of variations were 1.7% and 2.7% for quality check high and low, respectively. Between run coefficients of variations were 1.9% to 8.3% for 5 samples covering the measuring range tested over 5 days in triplicate. Samples were analyzed in batches, randomized, in single determinations, and calibration, and quality check were performed per IUO IFU. The measuring range of the test is between 50 and 3000 ng/ml; samples with higher concentrations were retested per automatic rerun function in a 1:15 dilution.

### Adjudication of AKI

Three independent adjudicators assessed clinical data to determine if a subject fulfilled the criteria for stage 2/3 AKI at 48- to 72-hours after ICU admission. The adjudicators developed an adjudication plan independently of the study sponsor and investigators (Supplementary Figure S1). The adjudicators were not informed of uNGAL results at any time during the study or the adjudication process. The adjudicators assessed different baseline SCr concentration criteria to make a final assessment based on available data: lowest documented SCr value in the 90 days before ICU admission (if available), imputed SCr based on the modified Schwartz criteria (as has been validated in the pediatric AKI literature),^18^ or 3 documented SCr concentrations chosen by the site principal investigator within 30 days before ICU admission (if available). The last option of 3 SCr values in the previous month was used for the GUIDANCE study only. Each case was assigned initially and randomly to 2 of the 3 adjudicators. Each of the 3 adjudicators had a similar initial caseload. The case was finalized if both initial adjudicators agreed on the presence or absence of stage 2/3 AKI. Cases were referred to a third adjudicator for final determination if there was no agreement with the first 2 adjudicators.

### Statistical Analysis

The primary outcome was the presence of SCr-based KDIGO stage 2/3 AKI at 48- to 72-hours after ICU admission. A sufficient data set for analysis of the primary outcome was characterized by a uNGAL result in the first 24-hours of ICU admission and SCr results in the first 12-hours of admission, 24- to 48-hours, and 48- to 72-hours after ICU admission. Demographic variables are reported as means with SDs or medians with interquartile range depending on data normality distribution (Shapiro-Wilk test). Urine NGAL is reported as ng/ml and was not normalized for urine creatinine concentration. We assigned a uNGAL concentration value of 51 ng/ml for all results below the lower limit of detection of 50 ng/ml.

Urine NGAL performance characteristics within the first 24-hours of ICU admission to predict stage 2/3 AKI 48- to 72-hours after ICU admission were assessed by sensitivity, specificity, positive and negative predictive values, and AUC-ROC. The optimal NGAL cutoff was determined by Youden Index (J-statistic) with a preference for specificity. All point estimates included 95% confidence intervals. Although GUIDANCE was not powered for subgroup analyses, we performed sensitivity analyses of uNGAL performance for biological sex based on external anatomy, imputation of baseline SCr method, and for each of the 4 eligibility criteria. We also assessed uNGAL performance characteristics for those who did or did not have SCr values indicative of AKI stage 2/3 on admission to the ICU. Specifically, assess the performance of urinary NGAL in the first 24 hours of ICU admission to predict stage 2/3 AKI development (in the case of SCr values not indicative of stage 2/3 AKI presence at admission) or stage 2/3 AKI persistence (in the case of SCr values indicative of stage 2/3 presence on admission). Intergroup rates are compared using χ^2^ analysis. Student *t*-test or Mann-Whitney U test were used to compare means or medians based on continuous variables based on normality of data distribution. Primary statistical analyses were performed using R-statistics version 4.1. A *P*-value of < 0.05 was considered statistically significant for inter-group analyses.

## Results

### EARNEST

**Figure 1:**
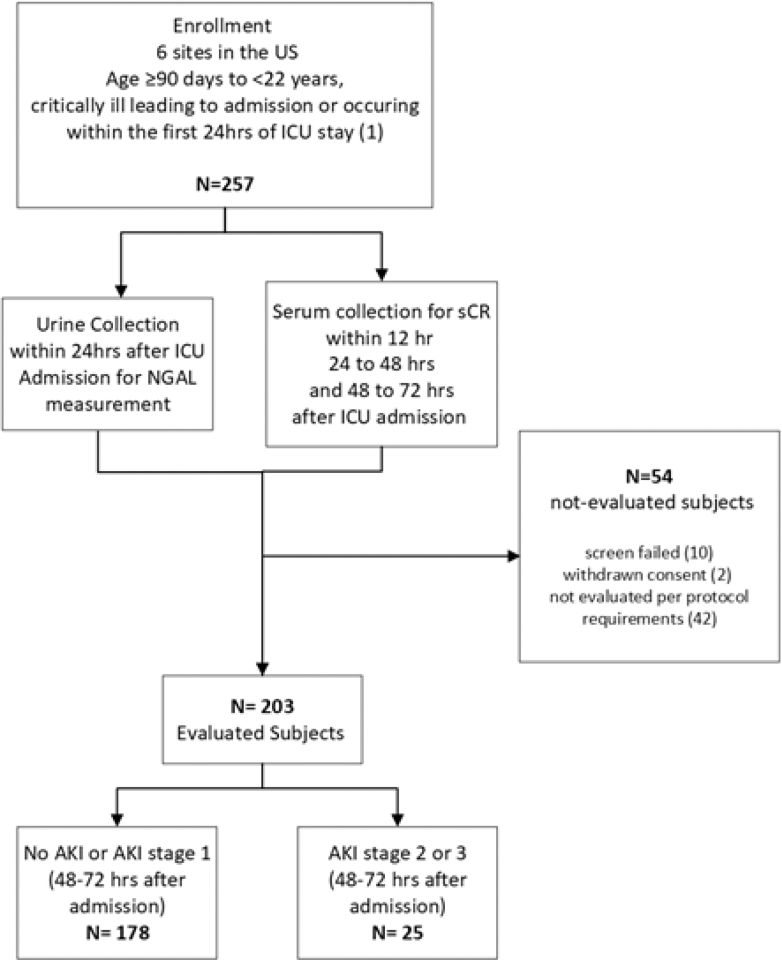
Design of the EARNEST study of pediatric subjects who are critically ill. Critical illness was defined as mechanical ventilation, vasoactive medication administration, history of solid organ transplantation (kidney transplantation included only if > 3 months prior), history of bone marrow/stem cell transplantation, or hypotension, defined as having received ≥ 40 ml/kg of resuscitative fluid in pre-ICU within 6-hours before or in first 12-hours of ICU admission. Subjects excluded from analysis were due to screen failure (not meeting inclusion/exclusion criteria), withdrawn consent, not evaluable per protocol requirement, including missing urine or SCr values, as well as improper handling, storage, processing, or insufficient volume of samples.

A total of 257 subjects were enrolled, with 203 subjects (79.0%) determined to be evaluable for the primary objective per the protocol (Figure 1). Mean age of the evaluable EARNEST subjects was 9.7 ± 6.0 years (range 3 months to 21 years) and 92 were female (45.3%). The remaining 54 subjects (21.0%) were screen-failed (10), withdrawn (2), or not evaluated per protocol requirements (42). All 203 evaluable subjects (111 male [54.7%]) were adjudicated for AKI staging.

Twenty-five EARNEST subjects (12.3%) had stage 2/3 AKI at 48- to 72-hours after ICU admission per adjudication (8 of whom had SCr concentrations indicative of stage 2 or 3 AKI at pediatric ICU admission). The AUC-ROC for uNGAL performance was 0.72 (95% CI: 0.62–0.84). The J-statistic was > 0.35 for cutoffs between 114 and 150 ng/ml, therefore an intermediate uNGAL concentration of 125 ng/ml was selected as the preferred cutoff for GUIDANCE. Urine NGAL clinical performance characteristics based on this 125 ng/ml cut point are detailed in Table 1.

**Table 1:** EARNEST and GUIDANCE uNGAL performance characteristics using a 125 ng/ml cutoff to predict KDIGO stage 2/3 AKI at 48- to 72-hours of ICU admission

Performance characteristic	aEARNEST	aGUIDANCE
Sensitivity	56.0% (34.9%–75.6%)	72.3% (57.4%–84.4%)
Specificity	82.0% (75.6%–87.4%)	86.3% (82.4%–89.3%)
Positive predictive value	30.4% (21.5%–41.1%)	34.7% (28.5%–41.5%)
Negative predictive value	93.0% (89.5%–95.4%)	96.9% (95.1%–98.0%)
AUC	0.73 (0.62–0.84)	0.83 (0.76–0.90)

AUC, area under the curve.

a All values are point estimate (95% confidence interval)

### GUIDANCE

**Figure 2:**
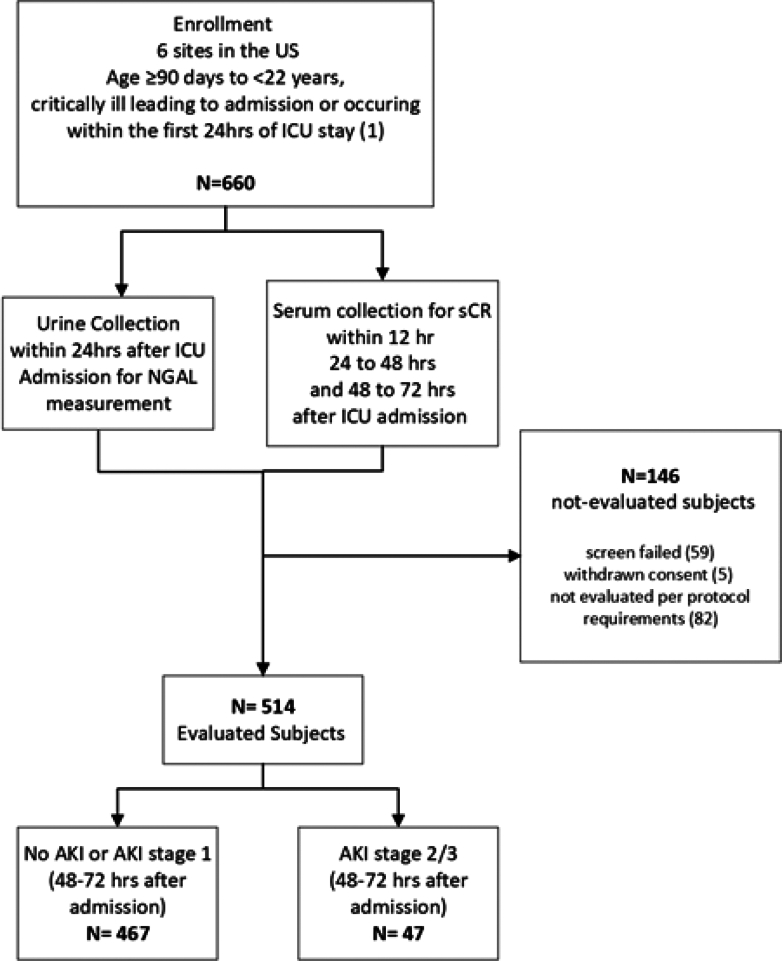
Design of the GUIDANCE study of pediatric subjects who are critically ill. Critical illness was defined as mechanical ventilation, vasoactive medication administration, history of solid organ transplantation (kidney transplantation included only if > 3 months prior), history of bone marrow/stem cell transplantation, or hypotension, defined as having received ≥ 40 ml/kg of resuscitative fluid in pre-ICU within 6-hours before or in first 12-hours of ICU admission. Subjects excluded from analysis were due to screen failure (not meeting inclusion/exclusion criteria), withdrawn consent, not evaluable per protocol requirement, including missing urine or SCr values, as well as improper handling, storage, processing, or insufficient volume of samples.

A total of 660 subjects were enrolled and 514 subjects (77.9%) were evaluated for the primary objective per the protocol (Figure 2). Of the 146 subjects (22.1%) who were not evaluated, 59 were screen-failed, 5 withdrawn, and 82 were not evaluated per protocol requirements. Subject demographics are presented in Table 2. In total, all 514 subjects, 243 female (47.3%) and 271 male subjects (53.7%) , were adjudicated. The mean age of the subjects was 9.1 ± 6.0 years (range 3 months to 21 years). One hundred seventy subjects (33.1%) met more than one of the admission reasons listed in the inclusion criteria.

**Table 2:** GUIDANCE subject clinical characteristics

GUIDANCE (*n* = 514)	
Sex (F/M)	243/271
Age (yrs)	9.1 ± 6.0
Weight (kilograms)	39.7 ± 27.8
Inclusion criteria (ICU admission reason)	
Invasive mechanical ventilation	343
Vasoactive medication	193
Fluid bolus	100
Stem cell transplantation	18
Solid organ transplantation	73
Met >1 inclusion criteria	170

F, female; ICU, intensive care unit; M, male.

Forty-seven GUIDANCE cohort subjects (9.1%) had stage 2/3 AKI at 48 to 72 hours after ICU admission. The adjudicators used measured SCr for baseline in 321 cases (62.4%) and imputed SCr for baseline in 193 cases (37.6%). The median time of urine collection for NGAL assessment was at 15.0 (9.4, 19.4) hours after ICU admission. The AUC-ROC for uNGAL performance was 0.83 (95% CI: 0.76–0.90). uNGAL clinical performance characteristics based on the 125 ng/ml cut point are detailed in Table 1.

We performed multiple subset analyses to assess the potential differences in uNGAL performance to predict KDIGO stage 2/3 AKI. We observed no differences in performance for subjects with AKI adjudication using a measured versus imputed baseline SCr (AUC-ROC: 0.83 [0.75–0.91] vs. 0.83 [0.70–0.96], *P* = 0.99) or biological sex (female: 0.85 [0.76–0.95]; male 0.82 [0.73–0.91], *P* = 0.63). Likewise, we observed no performance differences based on individual eligibility criteria (receipt of invasive mechanical ventilation [*P* = 0.13], vasoactive medication [*P* = 0.47], fluid bolus [*P* = 0.94] or presence of a stem cell/solid organ transplant [*P* = 0.27]) (Table 3). In addition, we found no performance differences for subjects who met only 1 versus more than 1 inclusion criteria (AUC-ROC: 0.81 [0.70–0.91] vs. 0.83 [0.74–0.91], *P* = 0.78).

**Table 3:** Comparison of test performance for each of the GUIDANCE inclusion criterion

Performance characteristic	Inclusion criteria							
	Fluid bolus		Invasive mechanical ventilation		Vasoactive medication		Stem cell or solid organ transplant^a^	
	Yes (100)	No (414)	Yes (343)	No (171)	Yes (193)	No (321)	Yes (91)	No (423)
AUC-ROC (95% CI)	0.84 (0.66–1.00)	0.83 (0.79–0.90)	0.80 (0.72–0.89)	0.90 (0.81–0.98)	0.84 (0.76–0.92)	0.79 (0.67–0.91)	0.87 (0.76–0.96)	0.80 (0.70–0.89)
*P*-value	0.94		0.13		0.47		0.27	

AUC-ROC, area under the curve of a receiver operator characteristics curve.

a Since there were only 18 patients with stem cell transplantation, they were combined with solid organ transplant patients as is typically done for risk stratification.^19^^,^^20^

Finally, we assessed performance characteristics of the uNGAL cutoff of 125 ng/ml for subjects who did or did not present with SCr values indicative of AKI stage 2/3 on the day of ICU admission. Ninety-two subjects (17.9%) presented with SCr values indicative of stage 2/3 AKI on admission, and 29 of these subjects (31.5%) had stage 2/3 AKI that persisted for 48- to 72-hours. Twenty-three of 41 subjects with uNGAL ≥ 125 ng/ml had persistent AKI, whereas only 6 of 51 subjects with uNGAL < 125 ng/ml had persistent AKI (56.1% vs. 11.8%, *P* = 0.001). Four hundred twenty-two subjects did not have SCr values indicative of stage 2/3 AKI on admission, and 18 of these subjects (4.3%) developed stage 2/3 AKI at 48 to 72 hours. Eleven of the 57 subjects (19.3%) without SCr indicative of stage 2/3 AKI on admission and uNGAL ≥ 125 ng/ml developed stage 2/3 AKI at 48 to 72 hours, compared with 7 of 365 subjects (5.2%) with uNGAL < 125 ng/ml (19.3% vs. 1.9%, *P* = 0.001). Overall performance characteristics for persistent or development of stage 2/3 AKI are depicted in Table 4.

**Table 4:** Urine NGAL 125 ng/ml cutoff performance characteristics for KDIGO stage 2/3 at 48- to 72-hours stratified by SCr values indicative of AKI presence or absence at ICU admission

Performance characteristic	aSCr indicative of stage 2/3 AKI on day of ICU admission (*n* = 92)	aSCr indicative of no stage 2/3 AKI on day of ICU admission (*n* = 422)
Sensitivity	79.3% (60.3–92.0%)	61.1% (35.8–82.7%)
Specificity	71.4% (58.6–82.1%)	88.6% (85.1–91.5%)
Positive predictive value	56.1% (45.3–66.2%)	19.3% (13.1–27.4%)
Negative predictive value	88.2% (78.3–94.0%)	98.1% (96.6–98.9%)

AKI**,** acute kidney injury; ICU, intensive care unit; SCr, serum creatinine.

a All values are point estimate (95% confidence interval).

## Discussion

We performed prospective cutoff derivation and validation studies of uNGAL performance in the first 24-hours of ICU admission to predict KDIGO stage 2/3 AKI presence at 48 to 72 hours after ICU admission. Overall, uNGAL performed very well (AUC-ROC 0.83) to identify risk of AKI in a high-risk population (as defined by the inclusion criteria). A cutoff of 125 ng/ml demonstrated excellent specificity and negative predictive value for stage 2/3 AKI presence at 48 to 72 hours. Importantly, this performance was maintained in every subanalysis performed (all with AUC-ROC of ≥ 0.79) based on inclusion criteria, use of imputed versus measured baseline SCr or biological sex in the GUIDANCE validation study. Finally, the 125 ng/ml cutoff value also demonstrated good sensitivity and specificity for persistent stage 2/3 AKI from admission to about 48–72-hours and excellent negative predictive value for subjects without a SCr indicative of stage 2/3 AKI at admission.

The data herein provide further evidence for uNGAL utility to distinguish between functional (formerly “pre-renal”) and structural (formerly “intrinsic”) AKI in a manner that the traditional functional markers of SCr and urine output cannot. Indeed, it has been nearly a decade since the Acute Disease Quality Initiative proposed a 2 × 2 table to categorize AKI based on functional marker and/or tubular injury marker positivity.^7^ This insight was catalyzed by an observation that patients with “subclinical” AKI (i.e., damage marker positive but functional marker negative) had similar morbidity and mortality rates to those who met the KDIGO criteria with a negative damage marker.^17^ In fact, this concept was assessed most directly in children after cardiac surgery,^21^ in which patients with functional (increased serum Cystatin C but normal uNGAL) AKI were more likely to have transient SCr elevation but those with subclinical AKI were more likely to have prolonged SCr elevation. A subsequent pediatric study recapitulated this pattern in children admitted to a non-cardiac ICU who were critically ill.^22^ As a result of these studies and others, Acute Disease Quality Initiative has recently proposed integrating stress or damage biomarkers with specific cutoffs into the KDIGO AKI diagnostic criteria.^23^ We do note, however, that we observed 18 of 41 patients with uNGAL ≥125 ng/ml did not have stage 2/3 AKI at 48- to 72-hours, (i.e. were “false positive”). It is possible that the presence of malnutrition or sepsis can negatively influence SCr increase necessary for AKI diagnosis, but this speculation requires additional study.

Given the consistently demonstrated good-to-excellent damage biomarker performance to predict AKI development and severity before changes in functional markers, the slow adoption of these diagnostic tests into clinical practice may seem puzzling, which has itself been the subject of public commentary^24^, ^25^, ^26^ over the past decade. Successful translation of diagnostics into the clinical setting requires demonstrating how test results can be used to guide clinical decision-making to improve patient outcomes.^24^ Fortunately, in the last few years, novel AKI biomarkers have been integrated into clinical decision support algorithms, using elevations above cutoffs to risk stratify patients for AKI and alter therapeutic interventions. The cell cycle arrest biomarkers TIMP2•IGBP7 (NephroCheck, Biomerieux, Inc) have been used to direct clinical bundles to optimize hemodynamics and limit nephrotoxic medication exposure and have led to decreased AKI development rates and severity in adults after cardiac surgery^27^ and noncardiac surgery.^28^ More recently, uNGAL has been integrated with the renal angina index AKI risk stratification system to guide fluid administration and continuous renal replacement therapy (CRRT) initiation in children who are critically ill.^29^ In this study, CRRT was considered for patients with a renal angina index ≥ 8 and a uNGAL ≥ 150 ng/ml when positive fluid accumulation reached 10% to 15% of the weight at ICU admission. Implementation of the pathway led to earlier initiation of CRRT, decreased fluid accumulation before CRRT initiation, shorter CRRT duration, decreased total and post-CRRT ICU length of stay, and improved ICU survival for patients who survived CRRT.

Our studies have several strengths. First, they were prospective and multicenter in design. Second, independent AKI adjudication was performed based on clinical course information in addition to SCr data, without knowledge of the uNGAL result. Third, the derived uNGAL cutoff of 125 ng/ml was validated in the second study, and we found no deterioration in predictive performance in any of the multiple subgroup analyses that we performed. Fourth, the KDIGO stage 2/3 AKI event rate of 9.1% is similar to that seen in other pediatric ICU studies,^4^^,^^30^, ^31^, ^32^ suggesting that our studies are representative of AKI epidemiology in pediatric ICUs at large. Finally, the studies were of equal size with similar performance results seen in the adult studies of the cell cycle arrest AKI biomarkers,^33^ which served as the clinical study data for Food and Drug Administration clearance of these biomarkers. To this end, EARNEST and GUIDANCE served as the clinical study data leading to its own Food and Drug Administration clearance for children aged ≥ 3 months to < 22 years NGAL in December 2023.^34^

We do acknowledge the limitations of our studies. First, 37% of the AKI adjudication relied upon imputed baseline, and not measured, SCr values. However, lack of a measured SCr result is common (usually ∼50%) in pediatric AKI studies, and imputation, with a validated method is necessary.^4^^,^^18^ Sensitivity analysis mitigates this concern with similar results for different methods of baseline SCr. Second, although representative of pediatric ICUs as noted above, the stage 2/3 AKI event rate is relatively low compared with what is observed in adults who are critically ill. Thus, the resultant statistical impact is seen in wide 95% CIs, especially for sensitivity and positive predictive values. Third, although we excluded patients with known CKD stage 4 or 5 from enrollment because baseline CKD can result in elevated baseline NGAL concentrations, our inclusion of patients with known CKD stage 3 could lead to some confounding because CKD Stage 3 has been associated with elevated baseline NGAL concentrations in elderly patients who developed AKI after iodinated contrast administration (mean 150.8 ± 77.8 ng/ml).^35^ Fourth, the baseline SCr at ICU admission may already represent ongoing AKI that initiated in the community setting. A uNGAL concentration that had already peaked and then decreased before ICU admission could lead to a false negative NGAL result. We believe this potential concern is mitigated by standard medical practice to follow SCr-based AKI at admission for persistence or resolution. Fifth, it is possible that the patients with a uNGAL of ≥125 ng/ml could develop AKI after 72-hours, but our study was not designed to assess for AKI after the 72-hour window. Finally, the studies only assessed uNGAL predictive performance over the first 72-hours of ICU admission, and this restricted timeframe likely underrepresents AKI epidemiology over the ICU time course. Nevertheless, we emphasize caution in extrapolating our data, and specifically the 125 ng/ml cut point, to later time courses during an ICU admission or outside of the ICU setting.

In conclusion, our prospective pediatric multicenter derivation and validation studies demonstrate a uNGAL concentration in the first 24-hours performs very well to predict KDIGO stage 2/3 AKI at 48- to 72-hours into an ICU course. We further suggest that a uNGAL cut point of 125 ng/ml can aid in the risk assessment for stage 2/3 AKI persistence of development, and when integrated with clinical assessment, lead to more personalized management of the child who is critically ill.

## Disclosure

CAB was the Chief Medical Officer of BioPorto Diagnostics, Incorporated during the conduct of these studies. SLG and RKB received consulting fees from BioPorto Diagnostics to develop the study during the submission of the clinical study report to the US Food and Drug Administration. SLG, RKB and KG receive other consulting fees from BioPorto Diagnostics.

## Supplementary Material

Supplementary File (PDF) **Table S1.** Complete list of EARNEST and GUIDANCE exclusion criteria. **Figure S1.** EARNEST and GUIDANCE Independent AKI Adjudication Plan. **Supplementary References. STROBE Statement and Checklist.**

## Footnotes

Supplementary File (PDF)
**Table S1.** Complete list of EARNEST and GUIDANCE exclusion criteria. **Figure S1.** EARNEST and GUIDANCE Independent AKI Adjudication Plan.
**Supplementary References**

**STROBE Statement and Checklist**
