# Supplementary material for: Derivation and Validation of an Optimal Neutrophil Gelatinase-Associated Lipocalin Cutoff to Predict Stage 2/3 Acute Kidney Injury (AKI) in Critically Ill Children
Source: Kidney Int Rep. 2024 May 15;9(8):2443–52. doi: 10.1016/j.ekir.2024.05.010 (PMC11328761; doi:10.1016/j.ekir.2024.05.010)
Supplement: Supplementary File (PDF) — Table S1. Complete list of EARNEST and GUIDANCE exclusion criteria. Figure S1. EARNEST and GUIDANCE Independent AKI Adjudication Plan. Supplementary References. STROBE Statement and Checklist. [file mmc1.pdf]

Supplemental Material

Supplemental Table 1 – Complete list of EARNEST and GUIDANCE exclusion criteria

1. Subjects receiving Renal Replacement Therapy in the first 24 hours of ICU admission.
2. Subjects with a diagnosed active Urinary Tract Infection at the time of NGAL sampling.
3. Subjects with a known history of chronic kidney disease stages 4 and 5 as evidenced by a pre-enrollment estimated GFR of less than 30 mL/min/1.73m^2 and under the care of a pediatric nephrologist.
4. Subjects with known congenital anomalies of the kidney and urinary tract at the time of presentation to the PICU.
5. Subjects with any known urothelial, urological or kidney malignancies.
6. Subjects that have had any urologic procedure or urologic surgery immediately prior to admission to the PICU.
7. Subjects that have had surgical nephrectomy less than 3 months prior to admission.
8. Subjects admitted to the pediatric ICU immediately post-operatively or up to three months following surgical correction of congenital heart disease.
9. Subjects with uncorrected congenital heart disease. *This criterion does not include patients with isolated uncorrected ventricular septal defect (VSD), atrial septal defect (ASD), patent ductus arteriosus (PDA) and patent foramen ovale (PFO).*
10. Subjects that have been previously enrolled in this study.

Supplemental Figure 1 – EARNEST and GUIDANCE Independent AKI Adjudication Plan

Pediatric AKI Adjudication Flowchart (1)

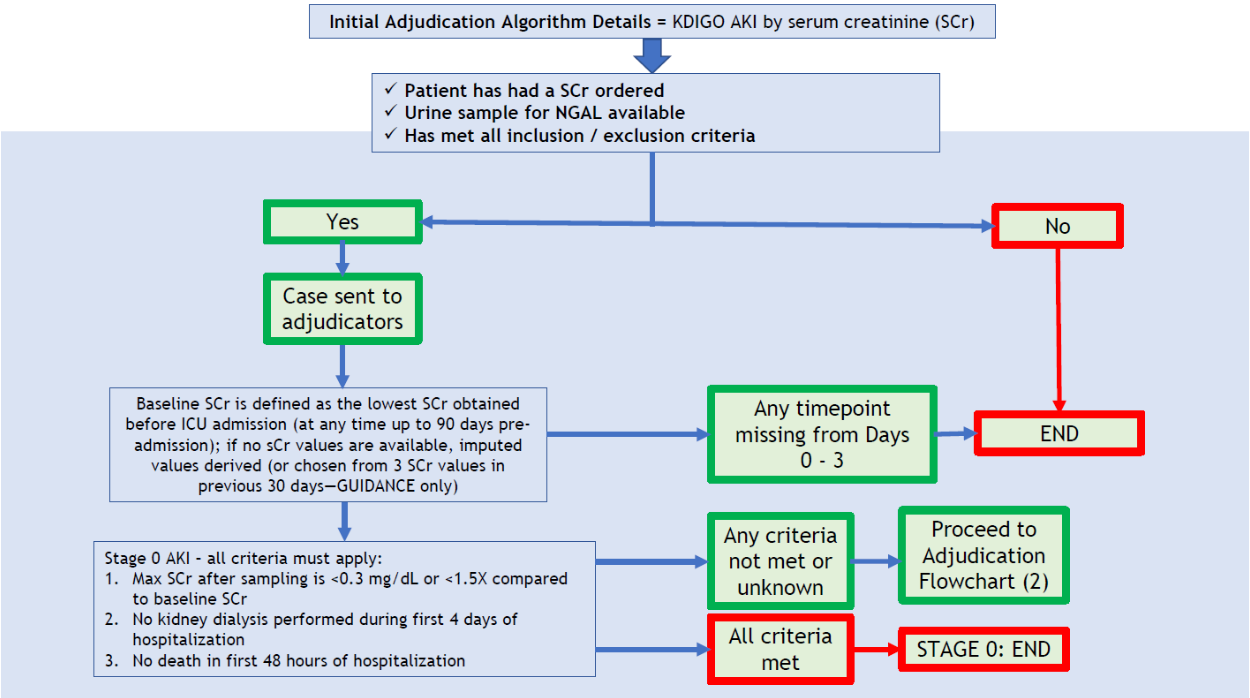

Pediatric AKI Adjudication Flowchart (2)

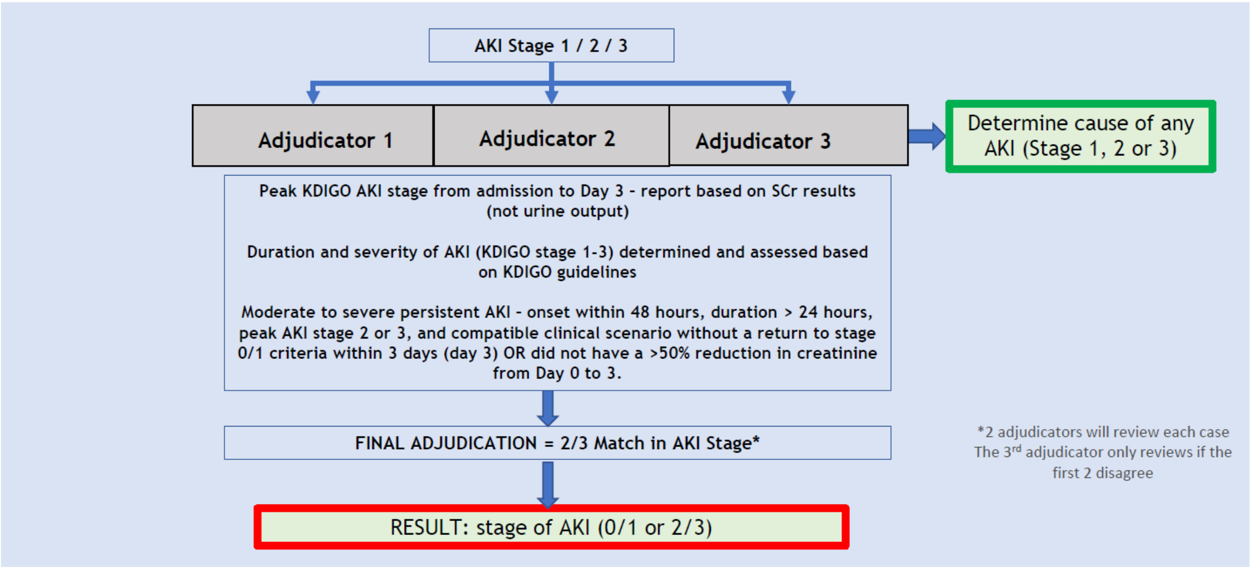

STROBE Statement—Checklist of items that should be included in reports of ***cohort studies***

	Item No	Recommendation	Page No
**Title and abstract**	1	(*a*) Indicate the study's design with a commonly used term in the title or the abstract<br>(*b*) Provide in the abstract an informative and balanced summary of what was done and what was found	(a) 4<br>(b) 5
**Introduction**			
Background/rationale	2	Explain the scientific background and rationale for the investigation being reported	6-7
Objectives	3	State specific objectives, including any prespecified hypotheses	7
**Methods**			
Study design	4	Present key elements of study design early in the paper	7
Setting	5	Describe the setting, locations, and relevant dates, including periods of recruitment, exposure, follow-up, and data collection	8
Participants	6	(*a*) Give the eligibility criteria, and the sources and methods of selection of participants. Describe methods of follow-up<br>(*b*) For matched studies, give matching criteria and number of exposed and unexposed	8
Variables	7	Clearly define all outcomes, exposures, predictors, potential confounders, and effect modifiers. Give diagnostic criteria, if applicable	9-10
Data sources/ measurement	8*	For each variable of interest, give sources of data and details of methods of assessment (measurement). Describe comparability of assessment methods if there is more than one group	9-10
Bias	9	Describe any efforts to address potential sources of bias	NA
Study size	10	Explain how the study size was arrived at	11
Quantitative variables	11	Explain how quantitative variables were handled in the analyses. If applicable, describe which groupings were chosen and why	11
Statistical methods	12	(*a*) Describe all statistical methods, including those used to control for confounding<br>(*b*) Describe any methods used to examine subgroups and interactions<br>(*c*) Explain how missing data were addressed<br>(*d*) If applicable, explain how loss to follow-up was addressed<br>(*e*) Describe any sensitivity analyses	11
**Results**			
Participants	13*	(a) Report numbers of individuals at each stage of study—eg numbers potentially eligible, examined for eligibility, confirmed eligible, included in the study, completing follow-up, and analysed<br>(b) Give reasons for non-participation at each stage<br>(c) Consider use of a flow diagram	12-13
Descriptive data	14*	(a) Give characteristics of study participants (eg demographic, clinical, social) and information on exposures and potential confounders<br>(b) Indicate number of participants with missing data for each variable of interest<br>(c) Summarise follow-up time (eg, average and total amount)	12-13
Outcome data	15*	Report numbers of outcome events or summary measures over time	12-13

Main results	16	(*a*) Give unadjusted estimates and, if applicable, confounder-adjusted estimates and their precision (eg, 95% confidence interval). Make clear which confounders were adjusted for and why they were included<br>(*b*) Report category boundaries when continuous variables were categorized<br>(*c*) If relevant, consider translating estimates of relative risk into absolute risk for a meaningful time period	12-13
Other analyses	17	Report other analyses done—eg analyses of subgroups and interactions, and sensitivity analyses	13-14
**Discussion**			
Key results	18	Summarise key results with reference to study objectives	14
Limitations	19	Discuss limitations of the study, taking into account sources of potential bias or imprecision. Discuss both direction and magnitude of any potential bias	16
Interpretation	20	Give a cautious overall interpretation of results considering objectives, limitations, multiplicity of analyses, results from similar studies, and other relevant evidence	17
Generalisability	21	Discuss the generalisability (external validity) of the study results	17
**Other information**			
Funding	22	Give the source of funding and the role of the funders for the present study and, if applicable, for the original study on which the present article is based	17

*Give information separately for exposed and unexposed groups.

Note: An Explanation and Elaboration article discusses each checklist item and gives methodological background and published examples of transparent reporting. The STROBE checklist is best used in conjunction with this article (freely available on the Web sites of PLoS Medicine at http://www.plosmedicine.org/, Annals of Internal Medicine at http://www.annals.org/, and Epidemiology at http://www.epidem.com/). Information on the STROBE Initiative is available at http://www.strobe-statement.org.
